# DNA Barcoding Mushroom Spawn Using EF-1α Barcodes: A Case Study in Oyster Mushrooms (*Pleurotus*)

**DOI:** 10.3389/fmicb.2021.624347

**Published:** 2021-05-17

**Authors:** Peng Zhao, Sen-Peng Ji, Xian-Hao Cheng, Tolgor Bau, Hong-Xin Dong, Xing-Xi Gao

**Affiliations:** ^1^Key Laboratory of Shandong Province for Edible Mushroom Technology, School of Agriculture, Ludong University, Yantai, China; ^2^Institute of Mycology, Jilin Agricultural University, Changchun, China

**Keywords:** intra- and interspecific variation, DNA barcode, mushroom spawn authenticity, oyster mushroom, sequence accessibility

## Abstract

Oyster mushrooms (genus *Pleurotus*) are widespread and comprise the most commonly cultivated edible mushrooms in the world. Species identification of oyster mushroom spawn based on cultural, morphological, and cultivated characteristics is time consuming and can be extraordinarily difficult, which has impeded mushroom breeding and caused economic loss for mushroom growers. To explore a precise and concise approach for species identification, the nuclear ribosomal internal transcribed spacer (ITS), 28S rDNA, and the widely used protein-coding marker translation elongation factor 1α (EF-1α) gene were evaluated as candidate DNA barcode markers to investigate their feasibility in identifying 13 oyster mushroom species. A total of 160 sequences of the candidate loci were analyzed. Intra- and interspecific divergences and the ease of nucleotide sequence acquisition were the criteria used to evaluate the candidate genes. EF-1α showed the best intra- and interspecific variation among the candidate markers and discriminated 84.6% of the species tested, only being unable to distinguish two closely related species *Pleurotus citrinopileatus* and *Pleurotus cornucopiae*. Furthermore, EF-1α was more likely to be acquired than ITS or 28S rDNA, with an 84% success rate of PCR amplification and sequencing. For ITS and 28S rDNA, the intraspecific differences of several species were distinctly larger than the interspecific differences, and the species identification efficiency of the two candidate markers was worse (61.5 and 46.2%, respectively). In addition, these markers had some sequencing problems, with 55 and 76% success rates of sequencing, respectively. Hence, we propose EF-1α as a possible DNA barcode marker for oyster mushroom spawn.

## Introduction

The pleurotoid fungi (*Pleurotaceae*, *Agaricales*, *Basidiomycota*), commonly known as oyster mushrooms, are widespread and comprise the most commonly cultivated edible mushrooms in the world. These fungi can also be used for various medicinal, environmental, and biotechnological purposes (Cohen et al., 2002; Kirk et al., 2008). Commercial production of *Pleurotus* spp. accounts for approximately 30% of the 15 million tons mushroom yield and has a value of more than US$50 billion (Chang, 2008), which reached approximately 63 billion in 2013 (Royse et al., 2017). The precise identification of mushroom spawn is crucial for mushroom breeding and professional mushroom cultivation.

In the majority of cases, due to the similarity of morphological traits, phenotypic plasticity under different cultivation substrates and environmental conditions, sometimes ambiguous and inconclusive mating tests (Bresinsky et al., 1976; Sánchez, 2004; Avin et al., 2012; Shnyreva and Shnyreva, 2015), and the lack of differential hyphal and mycelial characteristics, species identification of oyster mushroom spawn is time consuming and extraordinarily difficult, which has been an obstacle to mushroom breeding and caused economic loss for mushroom growers (Li et al., 2017). To address this issue, diffuse reflectance infrared Fourier transform (DRIFT) spectroscopy and molecular biology techniques have been employed (Yang et al., 2007; Huerta et al., 2010; Pawlik et al., 2012; Zervakis et al., 2012), but these techniques require much effort and can be inefficient. Moreover, different mycelium growth substrates had an effect on the outcome of species discrimination for the DRIFT method (Zervakis et al., 2012). Selecting an appropriate DNA barcode marker for the reliable and efficient species identification of oyster mushroom spawn is essential.

DNA barcoding is a powerful and rapid species identification tool using a standard short stretch of DNA (Hebert et al., 2003a). This approach is used to identify species of animals (Hebert et al., 2003a; Young et al., 2019), plants (Hollingsworth et al., 2009; Li et al., 2011), protists (Moniz and Kaczmarska, 2010; Pawlowski and Lecroq, 2010), and fungi (Seifert et al., 2007; Schoch et al., 2012; Lücking et al., 2020) for various purposes. For example, DNA barcoding contributes to food authenticity, and fraudulently labeled fish and mushrooms have been recognized (Filonzi et al., 2010; Carvalho et al., 2015; Zhao et al., 2017). The nuclear ribosomal internal transcribed spacer (ITS) was recommended as a universal DNA barcode marker for fungi (Schoch et al., 2012). The nuclear ribosomal 28S [large subunit (LSU)] rRNA gene sometimes distinguishes species on its own or when coupled with the ITS. The D1/D2 domain of 28S rDNA was adopted for delimitating species long before the DNA barcoding initiative for yeasts (Kurtzman and Robnett, 1998; Fell et al., 2000). The protein-coding gene translation elongation factor 1α (EF-1α) is also a potential barcode gene for *Fusarium* (Geiser et al., 2004) and is a precise barcode marker for *Trichoderma* and *Hypocrea* (Druzhinina et al., 2005).

In this study, ITS, 28S rDNA, and EF-1α were used as candidate markers to evaluate the feasibility of DNA barcode marker for oyster mushroom spawn. The intra- and interspecific variations (Hebert et al., 2004) and ease of sequence acquisition (Hollingsworth et al., 2009) were regarded as the criteria to determine the efficiency of the DNA barcode marker.

## Materials and Methods

### Materials

A total of 123 strains representing 13 species of the genus *Pleurotus* were sampled, including the type species *Pleurotus ostreatus* (Supplementary Table 1).

### DNA Amplification and Sequencing

The genomic DNA of each strain was extracted from mycelium grown on potato dextrose agar (PDA) (Wang and Zhuang, 2004). The nuclear rDNA ITS region (ITS1-5.8S-ITS2) and 28S rDNA were amplified and sequenced with the primer pairs ITS1 and ITS4 (White et al., 1990) and LROR and LR5 (Vilgalys and Hester, 1990; Rehner and Samuels, 1994), respectively. The partial EF-1α gene was amplified by the primer pair 728F and 1567R. The amplicon was sequenced with the primers EFjR and 1567Ra in addition to the amplification primer 728F (Carbone and Kohn, 1999; Rehner and Buckley, 2005), and the region between 728F and EFjR was analyzed. PCR was performed with a 2,720 Thermal Cycler (Applied Biosystems, Foster City, California, United States) using a 25-μl reaction system comprising 16 μl of double-distilled water, 2.5 μl of 10× PCR buffer, 2 μl of MgCl_2_ (25 mmol/L), 1.25 μl of each primer (10 μmol/L), 0.5 μl of dNTPs (10 mmol/L each), 1.25 μl of DNA template, and 0.25 μl of Taq DNA polymerase (5 U/μl). For ITS, the PCR conditions were an initial step of 5 min at 94°C, 30 cycles of 30 s at 94°C, 30 s at 53°C, and 30 s at 72°C, followed by 10 min at 72°C. For 28S rDNA, the PCR conditions were an initial step of 5 min at 94°C, 10 cycles of 30 s at 94°C, 30 s at 62°C (decreasing 1°C per cycle), 55 s at 72°C, plus 25 cycles of 30 s at 94°C, 30 s at 52°C, 55 s at 72°C, followed by 10 min at 72°C. For EF-1α, the PCR conditions were an initial step of 5 min at 94°C, 10 cycles of 30 s at 94°C, 55 s at 63 or 66°C (decreasing 1°C per cycle), 90 s at 72°C, plus 36 cycles of 30 s at 94°C, 55 s at 53, or 56°C, 90 s at 72°C, followed by 7 min at 72°C. The obtained amplicons were sequenced in both directions using an ABI 3730 XL DNA Sequencer (SinoGenoMax Co., Ltd.).

### Comparison of Intra- and Interspecific Divergences

The sequences were aligned using ClustalX 1.81 (Thompson et al., 1994) and manually edited to adjust the aligned sequences using BioEdit 7.0 (Hall, 1999). The aligned sequences of each gene were input into DNAStar 7.1.0 (Lasergene, WI, United States) to calculate the similarity matrix and to visually illustrate the intra- and interspecific variations of the candidate barcode markers for each of the 13 species tested in this study using the TaxonGap 2.4.1 software (Slabbinck et al., 2008).

### Evaluation of the Ease of Sequence Acquisition for the Tested Barcode Loci

The success rates were assessed for the PCR amplification and sequencing of the DNA barcode loci considered for oyster mushroom spawn. The criterion for successful amplification was when a single PCR band was obtained. The criterion for successful sequencing was a high-quality chromatogram. The success rate of PCR amplification multiplied by the success rate of sequencing to determine the overall success rate of PCR amplification and sequencing (Zhao et al., 2011a).

### Neighbor-Joining Tree Reconstruction

Neighbor-joining (NJ) trees inferred from ITS, 28S rDNA, and EF-1α gene sequences were reconstructed using MEGA 5.2 (Tamura et al., 2011) with the K2P model to show species divergence among the oyster mushroom spawn. Branch support was calculated by a bootstrap analysis with 1,000 replicates.

## Results

A total of 160 sequences of the candidate DNA barcode regions, ITS, 28S, and EF-1α, were analyzed from 13 *Pleurotus* species (Supplementary Table 1). To meet the requirements for a standard DNA barcode marker, the sequence lengths of all candidate loci are short. The fragments obtained were 488–578 base pairs (bp) for ITS, 804–829 bp for 28S, and 408–420 bp for EF-1α.

**FIGURE 1:**
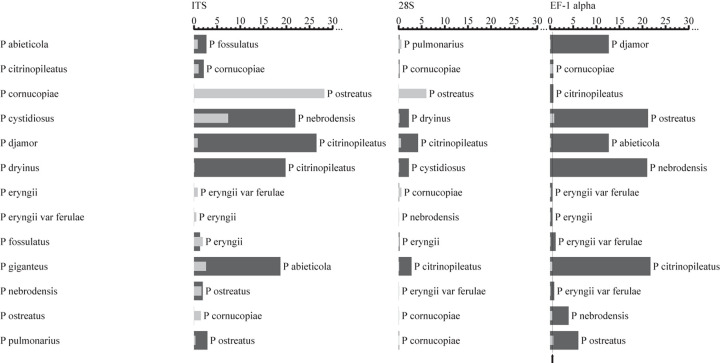
Comparisons of intra- and interspecific variations among the ITS, 28S rDNA, and EF-1α gene from the *Pleurotus* species tested. The gray and black bars represent the intra- and interspecific variations, respectively. The thin black lines indicate the smallest interspecific variation. The names next to the dark bars indicate the closest species.

The intra- and interspecific sequence variations of the candidate DNA barcode regions for each of the 13 species of *Pleurotus* mushroom spawn are shown visually in Figure 1, with the analyzed data in Supplementary Table 2. This result indicates that the EF-1α gene provided the best intra- and interspecific variation and the most species resolution power compared with the other markers (Figure 1). For the EF-1α gene, the sequences for the different strains of each included *Pleurotus* species were highly similar, and consequently, the intraspecific variations (indicated by the gray bars in Figure 1 and revealed by the maximum intraspecific distances of the tested 13 individual species in Supplementary Table 2) were low (the intraspecific sequence variation ranged from 0 to 0.9%, shown in Figure 1 and Supplementary Table 2). The smallest interspecific variation of all 13 species was 0.5%, as observed between *Pleurotus eryngii* var. *ferulae* and *P. eryngii* (Supplementary Table 2), which is shown as a thin black line in Figure 1. All species had intraspecific variations lower than 0.5% except for *Pleurotus pulmonarius*, *Pleurotus cystidiosus*, and *Pleurotus citrinopileatus* (Figure 1 and Supplementary Table 2). In addition to the 0.5% minimum interspecific variation mentioned above, the interspecific variations between *P. citrinopileatus* and *Pleurotus* cornucopiae (0.7%), *Pleurotus nebrodensis* and *P. eryngii* var. *ferulae* (0.9%), and *Pleurotus fossulatus* and *P. eryngii* var. *ferulae* and *P. eryngii* (1.2%), were all extraordinarily small (Supplementary Table 2).

The intra- and interspecific variations of ITS and 28S rDNA in the investigated oyster mushroom spawn were not appropriate because the intraspecific differences of several species were distinctly larger than the interspecific differences for both regions, which may lead to misidentification. For example, if ITS was used as a barcode marker, the intraspecific divergence for *P. cystidiosus* and *P. cornucopiae* is larger than the interspecific divergence between *P. citrinopileatus* and *P. cornucopiae* (Figure 1 and Supplementary Table 2).

The success rate of PCR and sequencing was the other criterion to evaluate the candidate barcode markers. A survey (Table 1) showed that the EF-1α gene was easily PCR amplified and sequenced, with success rates up to 84%. Unexpectedly, ITS and 28S rDNA had relatively low success rates of 55% and 76%, respectively.

**TABLE 1:** Success rates of PCR and sequencing of ITS, 28S rDNA, and EF-1α gene from 13 species of *Pleurotus* mushroom spawn.

Candidate barcode markers	ITS	28S rDNA	EF-1α
PCR	100%	100%	84%
Sequencing	55%	76%	100%
PCR and sequencing	55%	76%	84%

**FIGURE 2:**
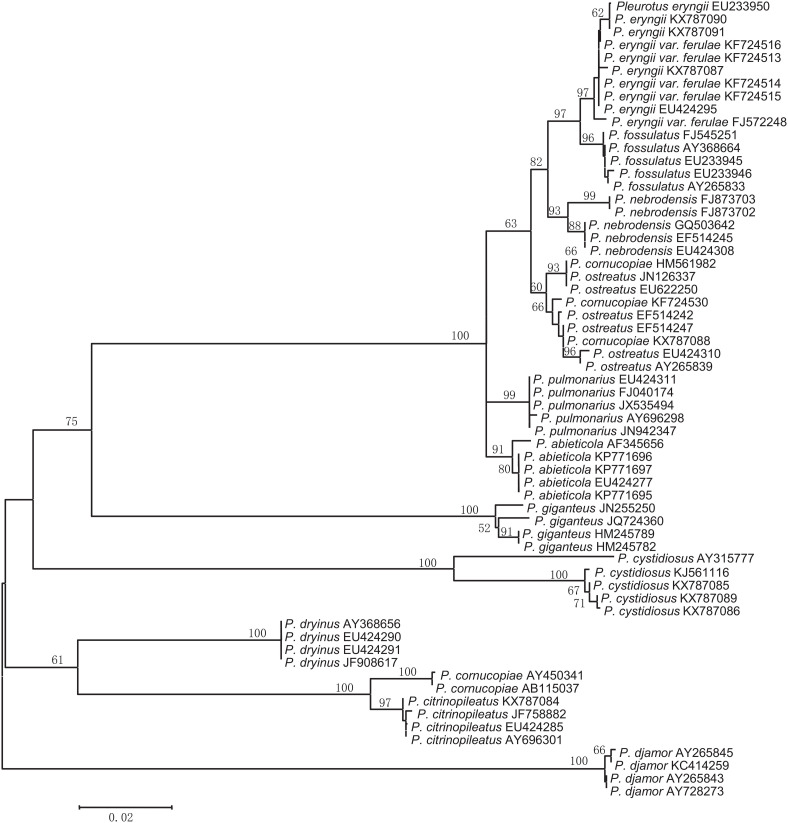
Neighbor-joining tree based on the ITS sequences from the *Pleurotus* species.

**FIGURE 3:**
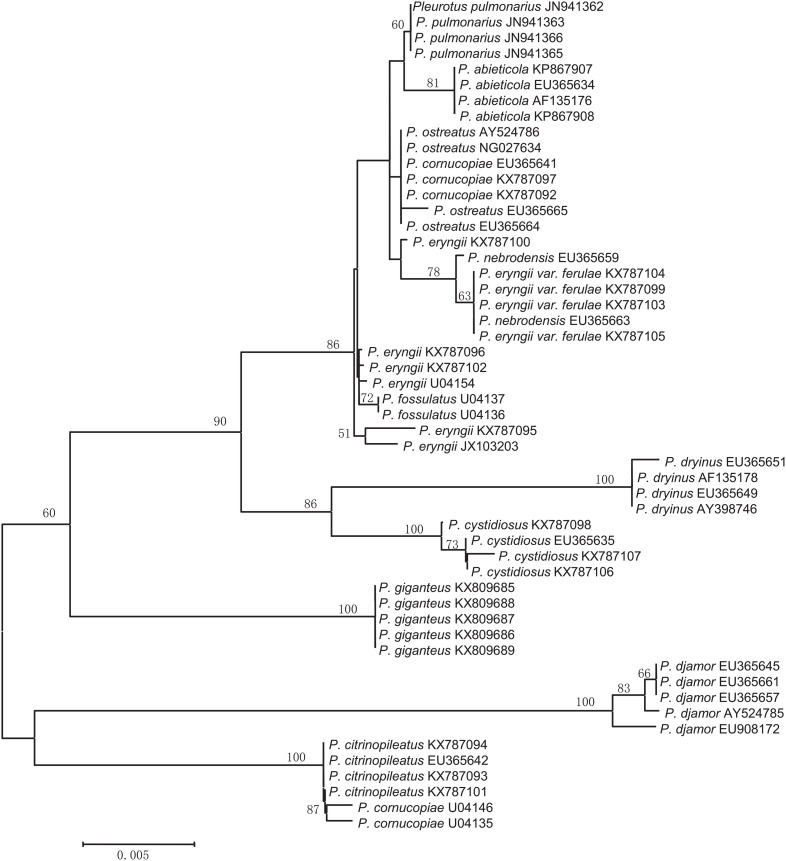
Neighbor-joining tree based on the 28S rDNA sequences from the *Pleurotus* species.

**FIGURE 4:**
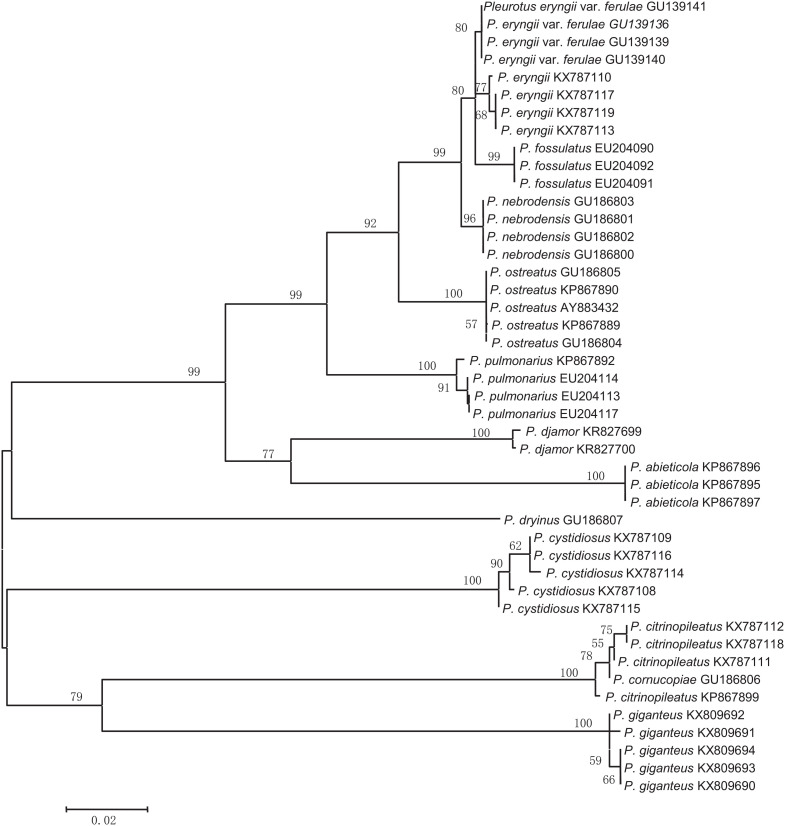
Neighbor-joining tree based on the EF-1α gene sequences from the *Pleurotus* species.

In most cases, species were separated from each other using the three NJ trees generated from the candidate genes (Figures 2–4). The species discrimination performance of the EF-1α gene was remarkable among the candidate regions, discriminating 84.6% (11 out of 13) of the species in the EF-1α-based NJ tree (Figure 4). Only *P. citrinopileatus* and *P. cornucopiae* could not be identified because they were highly cohesive. ITS discriminated 61.5% (8 out of 13) of the species (Figure 2), and 28S rDNA discriminated 46.2% (Figure 3).

## Discussion

According to the two criteria for assessing the suitability of DNA barcode loci, i.e., the intra- and interspecific variation and the sequence accessibility, the present study suggests that EF-1α can be considered a DNA barcode marker for oyster mushroom spawn.

EF-1α showed better intra- and interspecific variations among the candidate markers (Figure 1) and discriminated 84.6% of the species involved (Figure 4); this marker was only unable to distinguish *P. citrinopileatus* and *P. cornucopiae*. If these two species were considered to be one species, EF-1α could distinguish all of the species of *Pleurotus* in the present study. Actually, *P. cornucopiae* var. *citrinopileatus* is the synonym of the *P. citrinopileatus*^1^ which indicates that these two are closely related species. Ohira regarded *P. citrinopileatus* as a variety of *P. cornucopiae* (*P. cornucopiae* var. *citrinopileatus*) according to morphological similarities and mating test (Ohria, 1990), and the latter test was verified by Bao et al. (2004). The current study also supports the treatment of these two taxa as the same species. The species identification performance of EF-1α (84.6%) in this work was comparable with a previous identification rate of 89.5% in the genus *Neonectria* in Ascomycota (Zhao et al., 2011b); however, it was inferior to a previous 100% identification rate for the seven species of *Pleurotus* (Li et al., 2017), probably because of the greater sampling range in this study.

The four species *P. fossulatus*, *P. nebrodensis*, *P. eryngii* var. *ferulae*, and *P. eryngii* are closely related, as shown in Supplementary Table 2, which is in accordance with the fact that they were highly cohesive with a bootstrap value of 99% in the EF-1α-based NJ tree (Figure 4). Without considering the four interspecific pairwise distances for EF-1α between these four species, 0.5%, 0.7%, 0.9%, and 1.2% and the interspecific distance 0.7% between *P. citrinopileatus* and *P. cornucopiae*, the minimum interspecific variation was 4.0%, which occurred between *P. nebrodensis* and *P. ostreatus* (Supplementary Table 2). Assuming that 4.0% was chosen as the threshold of species discrimination, EF-1α could discriminate all of the tested species by a barcoding gap analysis, another species identification analysis, not by NJ tree but through clustering at a given threshold, which is based upon the hypothesis that all of the above distances are intraspecific, that is, *P. eryngii* var. *ferulae* and *P. eryngii* belong to a single species, as do the two species *P. citrinopileatus* and *P. cornucopiae*, and the four species *P. fossulatus*, *P. nebrodensis*, *P. eryngii* var. *ferulae*, and *P. eryngii*. Currently, *P. eryngii* var. *ferulae* is replaced by *P. eryngii*^2^, but the taxonomic status of the other four species remains unchanged. However, in this work, *P. citrinopileatus* is closely related to *P. cornucopiae* and the four species *P. fossulatus*, *P. nebrodensis*, *P. eryngii* var. *ferulae*, and *P. eryngii*. Undoubtedly, the taxonomy is complicated, and perhaps more work on *Pleurotus* is required. On the other hand, a conflict between current taxonomy and DNA barcoding often occurs (Seifert et al., 2007; Hollingsworth et al., 2009; Li et al., 2011; Zhao et al., 2011b; Schoch et al., 2012). In some cases, it results from candidate barcode genes, the sequence variations of which do not match the species due to their small variations (Zhao et al., 2011b). In contrast, sometimes DNA barcodes are associated with more species than accepted at that time, resulting in the situation wherein the cryptic species were contained (Hebert et al., 2004; Zhao et al., 2011b; Young et al., 2019).

Ideally, for a DNA barcode marker, all of the interspecific variations should exceed intraspecific ones, i.e., the minimum interspecific distance is larger than the maximum intraspecific distance, where the barcode gap exists, and barcoding is optimal when there is a one-to-one correspondence between one sequence and one species (Hebert et al., 2003a,b), namely, “One Species, One Barcode.” Therefore, a threshold could be set up to identify unknown organisms (Hebert et al., 2003a; Jeewon and Hyde, 2016), including oyster mushroom spawn or cultures. Otherwise, it will lead to misidentifications.

Another criterion for evaluating the suitability of DNA barcode gene is the acquisition of the barcode sequences. Although the success rate of sequencing for EF-1α was 100% in our study, that of PCR amplification was 84%. Nevertheless, EF-1α was the most likely to be acquired when compared with ITS and 28S rDNA (Table 1). Therefore, we propose EF-1α as the DNA barcode for oyster mushroom spawn. As shown in previous studies, EF-1α is the barcode gene for other fungal groups (Druzhinina et al., 2005; Zhao et al., 2011b).

In general, protein markers have higher species distinguishability, but PCR and/or sequencing failures sometimes occur (Schoch et al., 2012), which was verified by the 84% PCR and sequencing success rate for EF-1α in our study (Table 1). Likewise, the β-tubulin gene, the largest subunit of RNA polymerase II (RPB1) and the second largest subunit of RNA polymerase II (RPB2) genes, could not be amplified and sequenced successfully using universal primers in the present study and were not included in the data processing.

Nuclear ITS, the standard barcode marker for fungi recommended by the Fungal Barcoding Consortium, does not function well as a barcode marker for species resolution in this study. The PCR success rate was up to 100%, but the sequencing success rate was merely 55%. This was the lowest among the candidate loci, which agrees with a previous study in which ITS also exhibited the lowest PCR and sequencing success rate in comparison with the other protein-coding genes EF-1α, RPB1, and RPB2 (Li et al., 2017). A possible reason is that ITS sequences have heterogeneity in various copies (Wang and Yao, 2005; Fell et al., 2007; Lindner and Banik, 2011; Li et al., 2013), whereas the majority of protein-coding genes are single copies. Moreover, the intraspecific differences of several species were distinctly larger than the interspecific differences. Accordingly, the species discrimination capacity of ITS was only 61.5%, which was inferior to EF-1α (84.6%) but superior to 28S rDNA (46.2%). The geographical origins of the ITS sequences are more abundant than that of the other candidate regions, which may be one of the reasons for the divergence of ITS sequences. The limit of ITS for identifying species agrees with previous statements in some other groups (Nilsson et al., 2008; Seifert, 2008; Lücking et al., 2020). Obviously, ITS is not qualified for use as a DNA barcode marker for the mushroom spawn of the genus *Pleurotus*.

Nuclear 28S rDNA is a favored phylogenetic marker among many taxonomic groups (Rehner and Samuels, 1994; Johnson and Vilgalys, 1998; Hopple and Vilgalys, 1999) and has been employed for species delimitation for yeasts, and thus is considered to be the barcode marker for yeasts (Seifert, 2009). Our study found that the intra- and interspecific sequence variations for 28S rDNA (Figure 1) led to the lowest species delimitation ability (46.2%) and had some sequencing problems (76% success rate), which is inconsistent with other studies (Schoch et al., 2012). Most likely, this is due to the properties of these organisms, and more work is needed. Overall, 28S rDNA failed to act as a DNA barcode marker for the oyster mushroom spawn.

Species identification is a prerequisite and critical step in all biological research and applications. Correct identification unlocks the information of each organism, such as its physiological and biochemical properties, ecological roles, and risks or benefits to humans (Seifert et al., 2007; Zhao et al., 2011b). Morphological methods have traditionally served as the footstone of taxonomy and species identification. However, morphology-based identification is usually time consuming especially for species with scarce diagnostic features (Filonzi et al., 2010; Zhao et al., 2011b, 2017). For the past 17 years, DNA barcoding has drawn great attention as a powerful species identification tool. DNA barcoding is an emerging technology for reliable and concise species identification in the food and dietary supplement industry to determine the authenticity of mushrooms, traditional Chinese medicine, herbal dietary supplements, tea, meat, yak jerky, and aquatic products such as fish, crustaceans, and mollusks (Stoeckle et al., 2011; Little and Jeanson, 2013; Shen et al., 2016; Wang et al., 2016; Raja et al., 2017; Yacoub and Sadek, 2017; Hou et al., 2018). DNA barcoding authentication has considerable merits, one of which is that recognition requires only a tiny piece of tissue, is not affected by damaged and incomplete tissue, such as in powdered mycelium samples (Raja et al., 2017), and does not have growth stage limitations.

In this study, the DNA barcode gene EF-1α showed the most clear intra- and interspecific variations and identified a majority of oyster mushroom spawn species, except for closely related species. EF-1α also had a relatively high PCR and sequencing success rate and therefore qualified as a barcode marker for rapid and precise identification of oyster mushroom spawn to guarantee mushroom spawn production and facilitate mushroom production and trade. Our study suggests that other mushroom spawn may be DNA barcoded. More efforts should be directed at further improving barcoding for close relatives and for the widest range of fungi, particularly by utilizing technological improvements in genome sequencing to exploit novel barcode markers. Additionally, an integrated (polyphasic) approach combining molecular and phenotype data to continuously and critically revise existing knowledge is necessary to achieve high-quality taxa.

## Data Availability Statement

The datasets presented in this study can be found in online repositories. The names of the repository/repositories and accession number(s) can be found in the article/Supplementary Material.

## Author Contributions

PZ and TB conceived the project. PZ and S-PJ performed the experiments. PZ, X-HC, H-XD, and X-XG provided the resources. PZ analyzed the data and wrote the manuscript. All authors read and approved the final manuscript.

## Conflict of Interest

The authors declare that the research was conducted in the absence of any commercial or financial relationships that could be construed as a potential conflict of interest.

## Footnotes

1 http://www.speciesfungorum.org/Names/SynSpecies.asp?RecordID=303973 (accessed on 31 October 2020).

2 http://www.indexfungorum.org/Names/NamesRecord.asp?RecordID=171113 (accessed on 31 October 2020).

## Supplementary Material

Supplementary Table 1 Materials used in this study.

Supplementary Table 2 Intra- and interspecific variations among the ITS, 28S rDNA, and EF-1α gene from the *Pleurotus* species tested.
