# Supplementary material for: DNA Barcoding Mushroom Spawn Using EF-1α Barcodes: A Case Study in Oyster Mushrooms (Pleurotus)
Source: Front Microbiol. 2021 May 17;12:624347. doi: 10.3389/fmicb.2021.624347 (PMC8176306; doi:10.3389/fmicb.2021.624347)
Supplement: Supplementary Table 1 — Materials used in this study. [file Table_1.docx]

Supplementary Material

**Supplementary Table 1** Materials used in this study

| **Species** | **Collection number or source ^a^** | **Geographical origin** | **GenBank accession number** | | |
| --- | --- | --- | --- | --- | --- |
|  |  |  | **ITS** | **28S rDNA** | **EF-1α** |
| *Pleurotus* *abieticola* R.H. Petersen & K.W. Hughes | HKAS 46100 | China | KP771695 |  | KP867897 |
|  | HKAS 45720 | China | KP771696 | KP867907 | KP867895 |
|  | HKAS 45570 | China | KP771697 |  |  |
|  | CBS 102498 | Russia | EU424277 | EU365634 |  |
|  | TENN 058284 | Russia | AF345656 |  |  |
|  | RHP6551.1 | Unknown |  | AF135176 |  |
|  | HKAS45507 | China |  | KP867908 | KP867896 |
| *P.* *citrinopileatus* Singer | ACCC 51261 | China | EU424285 | EU365642 |  |
|  | pl.a0017 | Unknown | JF758882 |  |  |
|  | HMAS 63344 | China | AY696301 |  |  |
|  | CGMCC 3.7352 | China | **KX787084^b^** | **KX787093** | **KX787111** |
|  | CGMCC 3.7353 | China |  | **KX787094** | **KX787112** |
|  | CGMCC 5.838 | China |  | **KX787101** | **KX787118** |
|  | HKAS 85965 | China |  |  | KP867899 |
| *P. cornucopiae* (Paulet) Rolland | WC 608 | Unknown |  |  | GU186806 |
|  | D383 | UK |  | U04146 |  |
|  | CGMCC 5.149 | China | **KX787088** | **KX787097** |  |
|  | D1166 | Germany |  | U04135 |  |
|  | ACCC 50234 | China |  | EU365641 |  |
|  | CGMCC 5.599 | China |  | **KX787092** |  |
|  | CCMSSC 03779 | Unknown | KF724530 |  |  |
|  | TENN 055191 | Austria | AY450341 |  |  |
|  | ATCC 42045 | Czechoslovakia | AB115037 |  |  |
|  | PSW3 | China | HM561982 |  |  |
| *P. cystidiosus* O.K. Mill. | CGMCC 5.369 | China | **KX787089** | **KX787098** | **KX787115** |
|  | CGMCC 3.7470 | China | **KX787086** |  | **KX787108** |
|  | CGMCC 3. 7467 | China | **KX787085** |  |  |
|  | Pl. a0014 | Unknown | KJ561116 |  |  |
|  | ATCC 28598 | South Africa | AY315777 |  |  |
|  | CGMCC 5.360 | China |  | **KX787107** | **KX787114** |
|  | ACCC 50089 | China |  | EU365635 |  |
|  | CGMCC 3.7471 | China |  | **KX787106** | **KX787109** |
|  | CGMCC 5.409 |  |  |  | **KX787116** |
| *P. djamor* (Rumph. ex Fr.) Boedijn | ASI 2172 | Unknown | AY265845 |  |  |
|  | IFO 31859 | Unknown | AY265843 |  |  |
|  | Yuanlin No. 1 | Unknown | AY728273 |  |  |
|  | Q3 | Unknown | KC414259 |  |  |
|  | CBS 665.85 | Unknown |  | EU365645 |  |
|  | CBS 667.97 | Japan |  | EU365661 |  |
|  | ACCC 50836 | China |  | EU365657 |  |
|  | d8 | China |  | AY524785 |  |
|  | DMC 162 | Unknown |  | EU908172 |  |
|  | HKAS 51745 | China |  |  | KR827699 |
|  | HKAS 57781 | China |  |  | KR827700 |
| *P. dryinus* (Pers.) P. Kumm. | CBS 278.90 | Netherlands | EU424290 |  |  |
|  | CBS 449.77 | Czech Republic | EU424291 |  |  |
|  | 7239 | Italy | JF908617 |  |  |
|  | CBS 481.72 | Germany | AY368656 | EU365649 |  |
|  | CBS 804.85 | Netherlands |  | EU365651 |  |
|  | F91/1116 | Unknown |  | AF135178 |  |
|  | DSMZ 15789 | Germany |  | AY398746 |  |
|  | MW-84 | Unknown |  |  | GU186807 |
| *P.* *eryngii* (DC.) Quél. | ATCC 90797 | Czechoslovakia | EU233950 |  |  |
|  | CGMCC 5.775 | China | **KX787090** | **KX787100** | **KX787117** |
|  | CGMCC 5.860 | China | **KX787091** | **KX787102** | **KX787119** |
|  | CBS 100.82 | Slovakia | EU424295 |  |  |
|  | CGMCC 3.8516 | China | **KX787087** | **KX787096** | **KX787110** |
|  | D625 | Unknown |  | U04154 |  |
|  | CGMCC 3.7397 | China |  | **KX787095** | **KX787113** |
|  | L317 | China |  | JX103203 |  |
| *P. eryngii* var. *ferulae* (Lanzi) Sacc. | PF4 | Greece | FJ572248 |  |  |
|  | CCMSSC 00465 | Unknown | KF724513 |  |  |
|  | CCMSSC 02145 | Unknown | KF724514 |  |  |
|  | CCMSSC 02424 | Unknown | KF724516 |  |  |
|  | CCMSSC02235 | Unknown | KF724515 |  |  |
|  | CGMCC 5.1025 | China |  | **KX787105** |  |
|  | CGMCC 5.1024 | China |  | **KX787104** |  |
|  | CGMCC 5.918 | China |  | **KX787103** |  |
|  | CGMCC 5.745 | China |  | **KX787099** |  |
|  | WC933 | Israel |  |  | GU139139 |
|  | WC969 | Italy |  |  | GU139136 |
|  | WC929 | Israel |  |  | GU139140 |
|  | WC955 | Italy |  |  | GU139141 |
| *P.* *fossulatus* Cooke | ATCC 52666 | India | EU233946 |  |  |
|  | ATCC 90801 | Unknown | FJ545251 |  |  |
|  | ATCC 52666 | Unknown | AY265833 |  | EU204091 |
|  | ATCC 62885 | Unknown | AY368664 |  | EU204092 |
|  | ATCC 58080 | India | EU233945 |  | EU204090 |
|  | D1822 | Afghanistan |  | U04137 |  |
|  | D1821 | Afghanistan |  | U04136 |  |
| *P.* *giganteus* (Berk.) Karun. & K.D. Hyde | K(M)57571 | Malaysia | JN255250 |  |  |
|  | P6 | China | HM245789 |  |  |
|  | CMU54-1 | Thailand | JQ724360 |  |  |
|  | XY3 | China | HM245782 | **KX809687** | **KX809692** |
|  | MY | China |  | **KX809685** | **KX809690** |
|  | ZZ | China |  | **KX809689** | **KX809694** |
|  | ZD | China |  | **KX809688** | **KX809693** |
|  | LS | China |  | **KX809686** | **KX809691** |
| *P.* *nebrodensis* (Inzenga) Quél. | 940 | Unknown | FJ873703 |  |  |
|  | 927 | Unknown | FJ873702 |  |  |
|  | P491-1 | Unknown | GQ503642 |  |  |
|  | HMAS 86357 | China | EF514245 |  |  |
|  | ACCC 50869 | Unknown | EU424308 | EU365663 |  |
|  | ACCC 51060 | Unknown |  | EU365659 |  |
|  | WC976 | Italy |  |  | GU186803 |
|  | WC980 | Italy |  |  | GU186802 |
|  | WC979 | Italy |  |  | GU186801 |
|  | WC777 | Italy |  |  | GU186800 |
| *P.* *ostreatus* (Jacq.) P. Kumm. | CGMCC 5.370 | China | EF514247 |  |  |
|  | NW424 | China | EU622250 |  |  |
|  | HMAS 76520 | China | EF514242 |  |  |
|  | JZB21010017 | Unknown | JN126337 |  |  |
|  | CBS 375.51 | Italy | EU424310 | EU365665 |  |
|  | NRRL 3824 | Unknown | AY265839 |  |  |
|  | d7 | USA |  | AY524786 |  |
|  | TENN 053662 | Austria |  | NG027634 | AY883432 |
|  | CBS 125.13 | Unknown |  | EU365664 |  |
|  | HKAS 84903 | Germany |  |  | KP867889 |
|  | HKAS 53480 | Germany |  |  | KP867890 |
|  | WC971 | Italy |  |  | GU186805 |
|  | WC739 | Unknown |  |  | GU186804 |
| *P. pulmonarius* (Fr.) Quél. | ATCC 62887 | Unknown | JX535494 |  |  |
|  | CBS 665.97 | Thailand | FJ040174 |  |  |
|  | CBS 100130 | Tasmania | EU424311 |  |  |
|  | HMAS 76474 | China | AY696298 |  |  |
|  | 171 | China | JN942347 | JN941366 |  |
|  | 178 | China |  | JN941362 |  |
|  | 177 | China |  | JN941363 |  |
|  | 172 | China |  | JN941365 |  |
|  | D1979.j | India |  |  | EU204113 |
|  | BM247257 | India |  |  | EU204117 |
|  | D1979.2 | India |  |  | EU204114 |
|  | HKAS 76382 | China |  |  | KP867892 |

^a^ ACCC = Agricultural Culture Collection of China, Beijing, China; ATCC = American Type Culture Collection, Bethesda, MD, U.S.A.; CBS = Centraalbureau voor Schimmelcultures, Utrecht, The Netherlands; CCMSSC = China Center for Mushroom Spawn Standards and Control, Beijing, China; CGMCC = China General Microbiological Culture Collection Center, Beijing, China; HKAS = Herbarium of Cryptogams, Kunming Institute of Botany, Chinese Academy of Sciences, Kunming, China; HMAS = Herbarium of Mycology, (Academia Sinica), Institute of Microbiology, Chinese Academy of Sciences, Beijing, China; IFO = Institute for Fermentation, Osaka, Japan; NRRL = The Northern Regional Research Laboratory, ARS Culture Collection, Peoria, U.S.A.; TENN = The University of Tennessee Herbarium, Knoxville, U.S.A.

^b^ GenBank accession numbers in boldface indicating the newly submitted sequences.

**Supplementary Table 2** Intra- and inter-specific variations among the ITS, 28S rDNA and EF-1α gene from the *Pleurotus* species tested

| **Object Name** | **Maximum intraspecific distances (%)** | **Minimum interspecific distances (%)** | **Closest Object(s)** |
| --- | --- | --- | --- |
| Biomarker: ITS | | | |
| *Pleurotus cystidiosus* | 7.4 | 21.9 | *P. nebrodensis* |
| *P. eryngii* | 0.8 | 0 | *P. eryngii* var. *ferulae* |
| *P. eryngii* var. *ferulae* | 0.5 | 0 | *P. eryngii* |
| *P. fossulatus* | 1.9 | 1.3 | *P. eryngii* & *P. eryngii* var. *ferulae* |
| *P. nebrodensis* | 1.6 | 1.9 | *P. ostreatus* & *P. cornucopiae* |
| *P. ostreatus* | 1.5 | 0 | *P. cornucopiae* |
| *P. cornucopiae* | 28.2 | 0 | *P. ostreatus* |
| *P. abieticola* | 0.8 | 2.7 | *P. fossulatus* |
| *P. pulmonarius* | 0.3 | 2.9 | *P ostreatus* & *P. cornucopiae* |
| *P. giganteus* | 2.6 | 18.7 | *P. abieticola* |
| *P. citrinopileatus* | 1.0 | 2.1 | *P. cornucopiae* |
| *P. dryinus* | 0 | 19.8 | *P. citrinopileatus* |
| *P. djamor* | 0.8 | 26.5 | *P. citrinopileatus* |
| Biomarker:28S | | | |
| *P. cornucopiae* | 6.0 | 0 | *P. ostreatus* |
| *P. citrinopileatus* | 0 | 0.2 | *P. cornucopiae* |
| *P. ostreatus* | 0.1 | 0 | *P. cornucopiae* |
| *P. pulmonarius* | 0.1 | 0.1 | *P. cornucopiae* & *P. ostreatus* |
| *P. abieticola* | 0.6 | 0.2 | *P. pulmonarius* |
| *P. eryngii* var. *ferulae* | 0 | 0 | *P. nebrodensis* |
| *P. nebrodensis* | 0.1 | 0 | *P. eryngii* var. *ferulae* |
| *P. fossulatus* | 0 | 0.2 | *P. eryngii* |
| *P. eryngii* | 0.6 | 0.1 | *P. cornucopiae* & *P. ostreatus* |
| *P. dryinus* | 0.1 | 2.2 | *P. cystidiosus* |
| *P. cystidiosus* | 0.2 | 2.2 | *P. dryinus* |
| *P. giganteus* | 0 | 2.8 | *P. citrinopileatus* |
| *P. djamor* | 0.5 | 4.2 | *P. citrinopileatus* |
| Biomarker:EF-1alpha | | | |
| *P. abieticola* | 0 | 12.7 | *P. djamor* |
| *P. djamor* | 0.2 | 12.7 | *P. abieticola* & *P. nebrodensis* |
| *P. eryngii* var. *ferulae* | 0 | 0.5 | *P. eryngii* |
| *P. eryngii* | 0.2 | 0.5 | *P. eryngii* var. *ferulae* |
| *P. fossulatus* | 0 | 1.2 | *P. eryngii* var. *ferulae* & *P. eryngii* |
| *P. nebrodensis* | 0 | 0.9 | *P. eryngii* var. *ferulae* |
| *P. ostreatus* | 0.5 | 4.0 | *P. nebrodensis* |
| *P. pulmonarius* | 0.7 | 6.1 | *P. ostreatus* |
| *P. cystidiosus* | 0.9 | 21.2 | *P. ostreatus* |
| *P. dryinus* | - | 21.0 | *P. nebrodensis* |
| *P. citrinopileatus* | 0.7 | 0.7 | *P. cornucopiae* |
| *P. cornucopiae* | - | 0.7 | *P. citrinopileatus* |
| *P. giganteus* | 0.5 | 21.7 | *P. citrinopileatus* |
